# Cytokine profiles and phenotype regulation of antigen presenting cells by genotype-I porcine reproductive and respiratory syndrome virus isolates

**DOI:** 10.1186/1297-9716-42-9

**Published:** 2011-01-18

**Authors:** Mariona Gimeno, Laila Darwich, Ivan Diaz, Eugenia de la Torre, Joan Pujols, Marga Martín, Shigeki Inumaru, Esmeralda Cano, Mariano Domingo, Maria Montoya, Enric Mateu

**Affiliations:** 1 Departament de Sanitat i d'Anatomia Animals, Facultat de Veterinària, Universitat Autònoma de Barcelona, 08193 Cerdanyola del Vallès, Spain; 2 Centre de Recerca en Sanitat Animal (CReSA), UAB-IRTA, campus UAB, Edifici CR, 08193 Cerdanyola del Vallès, Spain; 3 NARO National Institute of Animal Health, Kannondai, Tsukuba, Ibaraki, Japan

## Abstract

The present study examined the immunological response of antigen presenting cells (APC) to genotype-I isolates of porcine reproductive and respiratory syndrome virus (PRRSV) infection by analysing the cytokine profile induced and evaluating the changes taking place upon infection on immunologically relevant cell markers (MHCI, MHCII, CD80/86, CD14, CD16, CD163, CD172a, SWC9). Several types of APC were infected with 39 PRRSV isolates. The results show that different isolates were able to induce different patterns of IL-10 and TNF-α. The four possible phenotypes based on the ability to induce IL-10 and/or TNF-α were observed, although different cell types seemed to have different capabilities. In addition, isolates inducing different cytokine-release profiles on APC could induce different expression of cell markers.

## Introduction

Porcine reproductive and respiratory syndrome virus (PRRSV) is one of the major pathogens affecting the swine industry worldwide. Control of the infection has proven to be difficult because of the limited knowledge on the ways by which the virus is transmitted between herds and also because of the lack of fully and universally protective vaccines. One of the main obstacles for the development of efficacious vaccines against PRRSV is the very partial understanding of PRRS immunopathogenesis. A few years ago, several papers comprehensively described the adaptive immune response to PRRS and showed that after infection or vaccination with either European or American PRRSV strains, pigs develop a rapid humoral response devoid of neutralising antibodies (NA). For some not yet fully elucidated reasons, NA develop much later in the course of infection [1]. Cell-mediated immunity, measured as virus-specific interferon-gamma (IFN-γ) secreting cells (SC), has an erratic behaviour for several weeks after the onset of infection [2-4] showing afterwards a trend to increase and reach a steady state. To explain such a unique picture of the adaptive immune response against viral infection seems to rely mostly but not only, on the early events of the innate immune response [5,6].

Early studies [7] showed that PRRSV was unable to induce significant IFN-α responses in vivo or in vitro. Also, infection of porcine macrophages with PRRSV impaired or abolished the IFN-α responses against transmissible gastroenteritis virus that is known to be a potent IFN-α inducer. Later on, other authors [8] showed that different North American PRRSV (genotype-II) isolates differed in their sensitivity to IFN-α and in their capabilities for inducing this cytokine. These authors suggested that the inhibition of IFN-α responses by PRRSV may be mediated by post-transcriptional mechanisms of regulation. IFN-α is not the only cytokine that seems to be affected by PRRSV infection. Several papers showed that interleukin-10 (IL-10) may play a role in the regulation of the immune response in PRRSV infection both in vitro and ex vivo [1-3,9,10]. Nevertheless, it has also been reported that different strains may induce different IL-10 responses in PBMC [3] and, therefore, different outcomes of the infection and the resulting immune response could be expected after infection with different strains. In addition, American-type PRRSV isolates seem to be able to downregulate other important components of the early immune response of antigen presenting cells (APC), in particular major histocompatibility complex (MHC) expression [10,11] and CD80/86 [12].

Taking into account those previous reports, it has become evident that IFN-α and IL-10 are clear targets for studying the regulation of the immune response against PRRSV. However, the available evidences suggest that different strains may probably produce different cytokine release patterns [13].

In the present study, a large collection of PRRSV strains was chosen to study the in vitro effects of PRRSV in different types of APC, with particular emphasis on cytokine profiles and the regulation of immunologically relevant cell markers. The participation of APC in the immune response is crucial and the examination of cytokine responses may provide information valuable to understanding the polarisation or the characteristics of the adaptive immune response. Also, presentation of antigens by APC requires an adequate expression of some molecules, of which MHC-I and MHC-II will be directly involved in presentation to CD4 and CD8 T cells. However, other molecules such as CD80/86 will be involved in such a presentation. The coincident expression of the CD80/86 co-stimulatory molecule will be crucial for determining the outcome of the antigen presentation-recognition by Th cells. Other molecules that were examined in this study, such as CD163 are in turn directly implied in the process of entry and replication of PRRSV. It is thought that CD163 is responsible for the uncoating of PRRSV once inside the cell. Other cell surface markers such as CD14 or SwC3, SwC9 may indicate different states of maturation of the dendritic cells.

The data presented in this work show that different PRRSV isolates are able to induce different patterns of cytokines and may modulate with different intensity the expression of immunologically relevant molecules.

## Materials and methods

### Viruses

Thirty-nine PRRSV strains were used in this study (Table 1). This set of strains included genotype-I (European) isolates from 1991 to 2006 of which some were retrieved from a viral collection (n = 15) and others were freshly isolated from frozen (-80 C) serum (n = 9) or lung tissue (n = 15) that have yielded positive results for PRRSV by RT-PCR. Freshly isolated viruses were from Spain and Portugal and archive viruses were from different countries of Western Europe. No epidemiological relationship was known to exist between the different isolates. Isolation was done in porcine alveolar macrophages (PAM) obtained from two healthy pigs free from all major diseases including PRRSV, pseudorabies virus and classical swine fever virus. Additionally, all PAM batches were tested for porcine circovirus type 2 (PCV2), hepatitis E virus and torque-tenovirus (TTV) according to previously described PCR protocols [14-16]. Viral stocks were also tested for mycoplasma by PCR. Viral stocks were produced from passages n = 2, n = 3 or n = 4 in PAM and, for each strain, batches of virus were larger enough to assure that the same batch could be used in all the experiments performed with that isolate, avoiding thus the use of different viral batches of the same strain for different experiments or replicas. Viral titrations were performed by inoculation of serial dilutions of viral stocks in PAM and readings were done by means of the immunoperoxidase monolayer assay using monoclonal antibodies for ORF5 (clon 3AH9, Ingenasa, Madrid, Spain) and ORF7 (clon 1CH5, Ingenasa, Madrid, Spain) using a method reported before with minor modifications [17].

**Table 1:** Description of the 39 European PRRSV isolates used in the present study.

Isolate	Titre log (TCID_50_/mL)*	Year	Tissue	Isolate	Titre log (TCID_50_/mL)*	Year	Tissue
**2652**	4.4	2005	Serum	**2996**	5.4	2005	Lung
**2654**	4.9	2005	Serum	**2998**	4.8	2003	Lung
**2655**	4.9	2005	Serum	**3003**	5.0	1994	Serum
**2658**	4.6	2005	Serum	**3004**	4.8	1994	Serum
**2744**	6.2	1991	Serum	**3005**	5.1	1994	Serum
**2751**	5.4	2005	Serum	**3009**	4.9	2005	Lung
**2788**	6.0	2006	Serum	**3012**	5.5	1997	Serum
**2797**	5.2	1991	Serum	**3013**	4.9	2004	Lung
**2804**	5.7	1992	Serum	**3016**	6.4	1991	Serum
**2805**	6.8	1992	Serum	**3249**	4.8	1991	Serum
**2810**	4.6	1992	Serum	**3256**	4.8	2005	Lung
**2812**	4.7	1992	Serum	**3262**	5.1	2005	Lung
**2894**	5.8	1991	Serum	**3266**	7.0	1991	Serum
**2896**	5.7	1991	Serum	**3267**	6.9	2006	Serum
**2982**	4.7	2005	lung				
**2983**	4.7	2005	Serum				
**2986**	4.8	2005	Lung				
**2987**	4.9	2005	Lung				
**2988**	4.9	2003	Lung				
**2990**	5.5	2005	Lung				
**2991**	5.1	2006	Lung				
**2992**	4.9	2006	Lung				
**2993**	4.9	2006	Lung				
**2994**	5.0	2006	Lung				
**2995**	5.1	2004	Lung				

*Maximum yield of viable virus obtained in cell culture supernatants (72 h) for a given strain after inoculation of 1 × 10^7 ^porcine alveolar macrophages (10 mL cell culture medium) with every strain at any multiplicity of infection.

In order to examine the need for virus viability for the induction of cytokines, three of the isolates were re-tested in parallel before and after inactivation by heat (60°C, 60 min). Complete inactivation was verified by inoculation of the heat-treated viral suspensions in PAM, which were examined at 72 h post-inoculation for the cytopathic effect and presence of PRRSV by IPMA. Untreated viable virus was used to assess the adequateness of the PAM batches for titrations.

### Isolation of bone marrow hematopoietic cells (BMHC) and differentiation of bone marrow-derived dendritic cells

Bone marrow hematopoietic cells were isolated, using a method previously described by Summerfield et al. [18] with minor modifications, from femora and humera of two PRRSV seronegative 6-week-old piglets obtained from a herd historically free of PRRSV. The cells obtained were frozen until needed. Bone marrow-derived dendritic cells were derived by using the protocol reported by Carrasco et al. [19]; namely, BMHC were cultured (37°C; 5% CO_2_) in Petri dishes (1 × 10^6 ^cells/mL in 10 mL of culture medium) with derivation medium (DM), namely RPMI 1640 medium supplemented with 10% fetal calf serum (FCS) (Invitrogen, Prat del Llobregat, Spain), 40 mM/mL L-glutamine (Invitrogen), 100 u/mL polimixine (Invitrogen), 50000 IU penicillin (Invitrogen), 50 mg/mL gentamicin (Sigma, Madrid, Spain), 100 ng/mL recombinant porcine granulocyte-monocyte colony stimulating factor (rpGM-CSF) (R&D systems, Madrid, Spain). On the third day of culture, the exhausted culture medium was replaced with 10 mL of fresh DM and, at day 6, half of the culture medium was replaced by fresh DM. Finally, at day 8 of culture, BMDC were collected by centrifugation and used in the assays.

### Obtaining and culturing of peripheral blood mononuclear cells (PBMC), SwC3^+ ^blood mononuclear cells and alveolar macrophages

Peripheral blood mononuclear cells and PAM were obtained from healthy pigs free from all major diseases as mentioned above. Peripheral blood mononuclear cells were separated from whole blood by density-gradient centrifugation with Histopaque 1.077 (Sigma). SwC3^+ ^(CD172a^+^) cells were purified from PBMC by positive selection using MACS Microbeads (Miltenyi Biotech SL, Pozuelo de Alcorcon, Spain). Briefly, the cells were incubated with mouse anti porcine CD172a-FITC (Serotec, Madrid, Spain) on ice for 30 min. After incubation, PBMC were washed and SwC3^**+ **^cells were coupled (15 min on ice) with anti-FITC magnetic particles (Miltenyi Biotec). Thereafter, PBMC were washed again and resuspended in MACS buffer (PBS plus foetal calf serum) and labelled cells were retrieved using LS selection columns (Miltenyi Biotec) according to the manufacturer's instructions. The purity of the cellular suspension was examined by flow cytometry analysis before further characterisation. The cell suspension obtained always had a richness of SwC3 ^+ ^≥ 92%.

Porcine alveolar macrophages were obtained by bronchoalveolar lavage of the lungs of piglets. After humane euthanasia, the lungs were removed aseptically and washed by infusion of PBS (Sigma) supplemented with 2% gentamicin through the trachea. The retrieved cell suspension was centrifuged (10 min at 450 *g*), washed and then the cells were frozen in liquid nitrogen until needed. Porcine alveolar macrophages were produced by adhesion to plastic of the retrieved cells. Parallel cultures of PAM were always examined for PRRSV and PCV2 by PCR as described above.

All these types of cells were cultured using RPMI 1640 medium supplemented with 10% FCS (Invitrogen, Madrid, Spain), 1 mM non-essential amino acids (Invitrogen), 1 mM sodium pyruvate (Invitrogen), 5 mM 2-mercaptoethanol (Sigma), 50000 IU penicillin (Invitrogen), 50 mg streptomycin (Invitrogen) and 50 mg gentamicin (Sigma). Trypan blue was used to assess viability.

### Cytokine profiles induced by different PRRSV strains

Four different types of cells were used: PBMC, PAM, peripheral blood SwC3^+ ^and BMDC. All cell types were cultured in supplemented RPMI as stated above. Peripheral blood mononuclear cells were cultured at a density of 5 × 10^6 ^cells/well in 1 mL of medium; PAM and SwC3^+ ^cells were cultured at 5 × 10^5 ^cells/well in 0.5 mL of medium and BMDC at 1 × 10^6 ^cells in 1 mL of culture medium. In preliminary experiments, the cells were stimulated with PRRSV at 0.1, 0.05 and 0.01 multiplicity of infection (m.o.i.) for 24 h. Since the profiles were not different in terms of positive/negative induction of a given cytokine, a 0.01 m.o.i. was chosen for final experiments using all 39 strains. All strains were examined three times (separate days), in triplicate cultures each time. For a given series of tests, all strains were tested in cells coming from the same animals. As a negative control, supernatants from mock-infected PAM were included. As positive controls, PHA (10 μg/mL) was used for PBMC and, LPS (10 μg/mL) and, gastroenteritis transmissible virus (m.o.i 0.01) for the other types of cells. Each time, cell culture supernatants of the three replicas were collected and mixed and the resulting mixtures were examined by ELISA to determine the concentrations of IFN-α, IL-10 and TNF-α (this cytokine was only examined in BMDC and SwC3^**+ **^cells). Also, for PAM, IL-1 and IL-8 were examined by means of commercial ELISA (R&D Systems). IFN-α capture ELISA was performed as reported previously [20] using K9 and F17 monoclonal antibodies. F17 was biotinylated (Phase Biotinylation Kit, PIERCE, Madrid, Spain). IFN-α recombinant protein (PBL Biomedical lab, Piscataway, New Jersey) was used as a standard. IL-10 capture ELISA was performed using commercial pairs of mAbs (swine IL-10, Biosource, Madrid, Spain) [2]. TNF-α capture ELISA was performed according to the manufacturer's instructions (Porcine TNF-α R&D systems). Cut-off of each ELISA was calculated as the mean optical density of negative controls plus three standard deviations. The values for the cytokine concentration in cell culture supernatants were calculated as a corrected concentration resulting from the subtraction of cytokine levels in mock-stimulated cultures from the values obtained for virus-stimulated cultures (concentration_PRRSV_-concentration _mock_).

### Phenotyping of bone marrow-derived dendritic cells before and after virus infection

Phenotypic characterisation of BMDC was done by means of flow cytometry at days 0 and +8 of the derivation process. Bone marrow-derived dendritic cells were further cultivated for 48 h more in DM without rpGM-CSF in the presence or absence (supernatants of mock-infected PAM) of different strains of PRRSV at 0.01 m.o.i and examined again. All strains were examined three times (separate days). The relative proportions of cells expressing SLA-I, SLA-II (DR), CD80/86, CD163, SwC3, SwC9, CD14 and CD16 were determined using mAbs 4B7, 2E9/13, mouse anti-porcine CD80 (Abyntek, Derio, Spain), 2A10/11, BL1H7, mouse anti-porcine SwC9-FITC (Serotec, Madrid, Spain), mouse anti-porcine CD14-FITC (Serotec), mouse anti-porcine CD16-FITC (Serotec). To further assess the effects of PRRSV infection, a double staining was performed for the SLA-II/CD80/86 and SLA-II/CD163 pairs. Four isolates were used for phenotype characterisation: isolate 3267 (IL-10^-^/TNF-α^-^), 3262 (IL-10^+^/TNF-α^+^), 3249 (IL-10^-^/TNF-α^+^) and 2988 (IL-10^+^/TNF-α^-^). In addition, cell culture supernatants (48 hours) of BMDC inoculated with PRRSV at 0.01 m.o.i were titrated in PAM as described above. In a second part of this experiment, the cells were incubated for 48 h with PRRSV isolates selected on the basis of their ability to induce cytokine release in BMDC: 3267 (IL-10^-^/TNF-α^-^), 3262 (IL-10^+^/TNF-α^+^), 3249 (IL-10^-^/TNF-α^+^) and 2988 (IL-10^+^/TNF-α^-^) and treated with a neutralising anti IL-10 antibody (0.15 μg/mL). After incubation, the cells were re-examined for changes in the phenotype.

### Statistical analysis

Statistical analyses were done using Statsdirect v.2.7.5. The comparison of the amounts of cytokines in different cell types was done using the Mann-Whitney test. The comparison of viral titres and cytokine levels was done by linear regression. The comparison of the results obtained in flow cytometry experiments (average and standard deviations) was performed using the Kruskal-Wallis test with multiple comparisons (Conover-Inman method). Statistical significance was set at *p *< 0.05.

## Results

### Cytokine profiles induced by different PRRSV strains

Examination of cell culture supernatants of PBMC yielded negative results for IFN-α for all PRRSV isolates. For IL-10, 9/39 strains induced the release of this cytokine (mean concentration 53 pg/mL; range 45-81 pg/mL) of which one was also positive for TNF-α. Eleven additional isolates also induced TNF-α in PBMC but not IL-10. Regarding PAM, all isolates were negative for IFN-α and 12 were positive for IL-10 but producing low levels (50 pg/mL; range 35-65 pg/mL) after correction. Also, all examined isolates induced high levels of IL-8 and IL-1 in PAM (on average > 8000 pg/mL for IL-8 and 326 ± 195 pg/mL for IL-1). For BMDC, seven strains were able to induce IL-10 release and 24 strains induced TNF-α; five strains were IL-10^+^/TNF-α^+ ^positive and 13 were double negative (Table 2). In SwC3^**+ **^cells, 21 strains induced IL-10 (comprising all but one of the BMDC IL-10 inducing strains) and 30 strains induced TNF-α (including all the TNF-α inducing strains for BMDC). Regarding IFN-α, all strains were negative but two that yielded borderline (close to cut-off) results in the ELISA. In any case, BMDC and SwC3^**+ **^cells were the more sensitive methods for the detection of these cytokine responses. Heat inactivation of the virus eliminated the capacity of the virus to induce the release of IL-10 and TNF-α but did not enhance IFN-α release. No differences were found regarding the amount of a given cytokine induced by strains in the same cell type; however, SwC3^**+ **^were higher producers of TNF-α than other cell types, particularly than BMDC (*p *< 0.05). Thus, when considering all strains producing TNF-α regardless of the IL-10 profile, average concentration of TNF-α in cell culture supernatants of SwC3^**+ **^cells was 1027 ± 775 pg/mL versus 575 ± 400 pg/mL for BMDC (p = 0.046). This difference was not seen for IL-10. No correlation was observed between the viral titer and the levels of a given cytokine.

**Table 2:** Distribution of 39 genotype-I PRRSV isolates according to their cytokine profiles.

	BMDC				SwC3^+^			
IL-10 pg/mL*	NA	NA	183 ± 137 (280-86)	410 ± 98 (522-297)	NA	NA	323 ± 259 (752-119)	501 ± 363 (1352-77)
TNF-α Pg/mL*	NA	502 ± 293 (1070-91)	NA	432 ± 300 (941-201)	NA	879 ± 715 (2,039-103)	NA	1321 ± 939 (2847-229)
**Total strains**	**13**	**19**	**2**	**5**	**5**	**13**	**4**	**17**

*Cytokine-inducing profiles for IL-10 and TNF-α were obtained using bone marrow-derived dendritic cells and sorted SwC3^+ ^peripheral blood mononuclear cells inoculated at a multiplicity of infection of 0.01. Values in the table show the average, standard deviation and range (pg/mL) of the concentrations in cell culture supernatants obtained for a set of strains inducing a given cytokine.

### Phenotyping of BMDC before and after virus infection

Freshly derived BMDC show the expected phenotype from the review in the literature and thus, this was a heterogenous population where about 30% of the cells expressed high levels of SLA-II (DR); 14% were CD163^+^; 31% were SwC3^+^, 52% were CD14^+ ^and 55% were CD16^+ ^(figures not shown). These cells were inoculated with different PRRSV strains selected considering the different cytokine-inducing phenotypes in BMDC or they were mock-inoculated with cell culture supernatants of PAM. After 48 h of incubation, mock-inoculated BMDC showed signs of maturation as evidenced by the increased proportion of cells expressing SLA-II, CD80/86, SwC3 and CD163 (data not shown).

**Figure 1:**
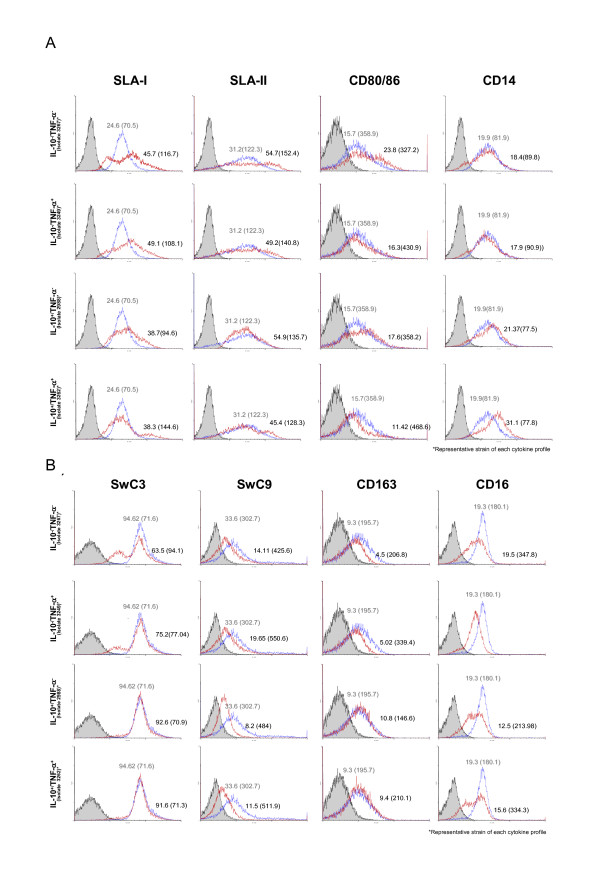
**Single colour flow cytometry analysis of changes in several immunologically relevant cell surface molecules of bone marrow-derived dendritic cells after 48 h of incubation with different PRRSV strains at a multiplicity of infection of 0.01 (as determined by titration in porcine alveolar macrophages)**. Grey histograms show cells stained with an irrelevant isotype-matched antibody and a secondary FITC or phycoerithrin conjugated antibody. Blue histograms show uninfected cells and red line histograms correspond to PRRSV-infected cells. Averages and variation coefficients are included. Grey values show the uninfected cell parameters and black values correspond to PRRSV-infected cells. A) SLA-I, SLA-II, CD80/86, CD14; B) SwC3, SwC9, CD163, CD16.

**Figure 2:**
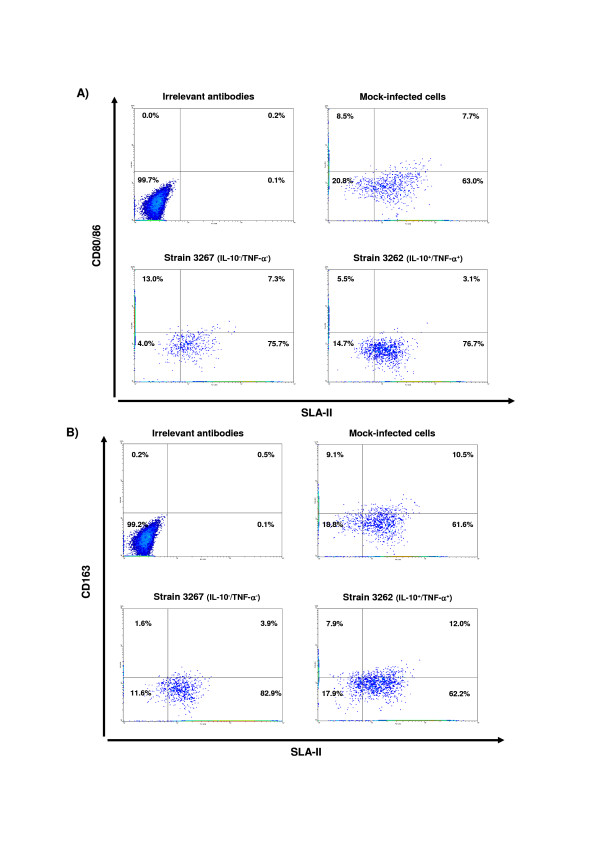
**Dual colour flow cytometry analysis of bone marrow-derived dendritic cells at 48 h post inoculation with different PRRSV isolates at 0.01 m.o.i.: A) SLA-II and CD80/86; B) SLA-II and CD163**.

Examination of BMDC 48 h after inoculation with the virus showed that strains with different cytokine-inducing properties regulated several cell markers differently (Figure 1 and Table 3). For SLA I, compared to mock-inoculated cells, strains 3267, 3249 and 3262 produced a decrease in the expression of that molecule while no changes were observed for strain 2988. For SLA-II, all strains but 3249 produced significant increases in expression (*p *< 0.03) compared to uninfected cells. For CD80/86, the behaviour of BMDC after infection differed depending on the strains. Interestingly, the double negative cytokine-inducing strain (3267) was the one that produced the highest increase in the expression of CD80/86 while the double positive strain (3262) produced a decrease in CD80/86 compared with the uninfected control (*p *= 0.01). For CD14, compared to the uninfected cells, the expression increased for strain 3262 (double positive cytokine-inducing strain), decreased for strain 3267 (double negative cytokine-inducing strain) and no changes were observed for strain 3249 and 2988. Regarding CD163, expression decreased only in the double negative strain (3267). When strain 3249 was used for stimulation, CD163 showed a trend for an increase although the p-value was not strictly significant (*p *= 0.09). Compared to uninfected cells, CD16 expression was always reduced upon PRRSV inoculation (*p *= 0.02) except for isolate 2988 and no differences were found for SwC9 and SWC3 expression. To further gain insight into these changes, SLA-II, CD80/86, and CD163 were examined in double staining experiments (Figure 2). The results show that strain 3262 (IL-10^+^/TNF-α^+^) promoted an increase in the expression of single SLA-II^+ ^(76% in inoculated cells versus 63% in mock-inoculated cells) simultaneously with a reduction of CD80/86 expression. It is worthy to note the decrease in the double positive subset percentage (7% in mock-infected cells; 3% in 3262-inoculated cells). In contrast, cells infected with 3267 (IL-10^-^/TNF-α^-^) exhibited an increase (75.7%) in the total proportion of SLA-II^+ ^single expressing cells but the numbers of SLA-II^-^/CD80/86^+ ^cells increased (from 8.5% in uninfected cells to 13.0% in 3267 infected cultures). For CD163, IL-10 inducing strains were able to keep the proportions of double positive SLAII/CD163 cells compared to mock-inoculated culture cells while infection with a IL-10^-^/TNF^- ^strain (3267) produced a clear decline of double positive SLAII/CD163 cells.

**Table 3:** Immunologically cell surface molecules in bone marrow-derived dendritic cells.

	Porcine reproductive and respiratory virus isolates used for stimulation of bone marrow-derived dendritic cells^#^					
**Molecule**	**Uninfected**	**3267**	**3249**	**2988**	**3262**	***p*-value**

SLA-I	98.2 ± 0.9^a^	93.9 ± 1.2^b^	93.5 ± 2.4^b^	98.3 ± 1.3^a^	94.0 ± 1.6^b^	*P *= 0.04
SLA-II	69.9 ± 3.5^a^	81.8 ± 3.4^b^	74.4 ± 1.7^a^	79.4 ± 4.0^b^	79.7 ± 3.8^b^	*P *= 0.03
CD80/86	15.2 ± 1.2^a^	29.4 ± 5.7^b^	13.4 ± 2.8^a^	15.9 ± 1.3^a^	10 ± 1.7^c^	*P *= 0.01
CD14	85.4 ± 2.2^a^	81.4 ± 1.3^b^	84.5 ± 1.3^a^	86.4 ± 1.3^a^	93.8 ± 0.3^c^	*P *= 0.03
SWC3	85.5 ± 5.8	75.5 ± 17.3	87.5 ± 9.7	81.4 ± 7.8	83.9 ± 2.7	*P *= 0.78
SWC9	52.2 ± 27.4	45.5 ± 5.6	42.3 ± 5.6	30.6 ± 1.6	33.6 ± 6.6	*P *= 0.24
CD163	19.7 ± 2.1^a^	4.9 ± 3.5^b^	24.6 ± 7.0^a^*	18.1 ± 4.7^a^	18.2 ± 1.5^a^	*P *= 0.03
CD16	88.8 ± 7.6^a^	65.8 ± 3.7^b*^	50.8 ± 15.6^c^	83.0 ± 2.4^a^	70.2 ± 0.7^b^	*P *= 0.02

^#^Average expression (three replicas) of some immunologically relevant cell surface molecules in bone marrow-derived dendritic cells. Each cell shows average percentage of cells expressing a given molecule and the standard deviation of the three observations. The comparison of the results was done using the Kruskal-Wallis test (*p*-value shown in the table) with multiple comparisons. Different superscript letters indicate statistically different (*p *< 0.05) percentages.

*p = 0.09

Titration in PAM of the cell culture supernatants produced in BMDC yielded titers of 10^6.5^, 10^5.5 ^and 10^5.0 ^TCID_50_/mL respectively for strains 3267, 3249 and 3262. These same strains yielded titers of 10^6.9^, 10^4.8 ^and 10^5.1 ^TCID_50_/mL when directly cultured in PAM under the same conditions, indicating no substantial differences between PAM and BMDC for supporting viral replication.

### Effects of IL-10 blocking

**Figure 3:**
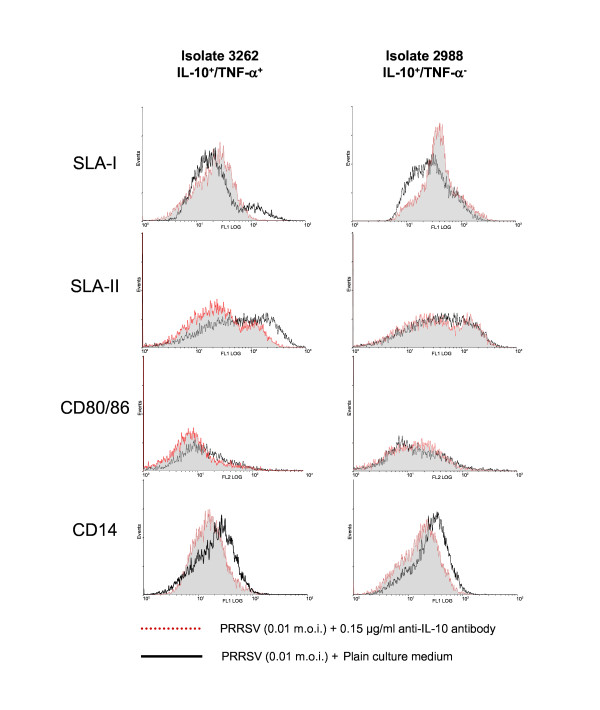
**Effect of IL-10 blocking in BDMC infected with two different PRRSV strains upon expression of SLA-I, SLA-II, CD80/86 and CD14**. Black solid lines represent the results obtained incubating BMDC with PRRSV in plain culture medium and red dotted lines represent the results obtained after IL-10 blocking.

As Figure 3 illustrates, blocking of IL-10 resulted in upregulation of the expression of SLA-I, downregulated CD14 and had no effect (strain 2988 IL-10^+^/TNF-α^-^) or downregulated (strain 3262 IL-10^+^/TNF-α^+^) CD80/86 and SLA-II.

## Discussion

Knowledge of interactions between PRRSV and APC is crucial to understand the unique features of the immunity and pathogenesis of PRRS. PAM are considered to be the main targets for PRRSV [17] although several reports [11-13,21-23] have shown that different DC may support replication of the virus, suffer a regulation of immunologically relevant cell surface proteins as well as produce cytokines upon infection with PRRSV. An accurate review of the scientific literature, however, reveals that every study intended to clarify the effects of the abovementioned interactions produced different results. Some papers indicated that PRRSV downregulates SLA-I and/or SLA-II while some others did not [11,12,21-23] or, for example, CD80/86 have been shown to be up and downregulated by PRRSV [11,12,21]. Similarly, some papers showed that IL-10 and TNF-α are cytokines produced by DC or PAM upon PRRSV infection, while some other papers reported the inability to detect these cytokines [9,11,12,21,23-26]. To say the least, this is an unusual picture for an animal virus. Thus, since cytokines are crucial elements in the regulation of the immune system and different works reported different findings regarding the ability of PRRSV in inducing cytokine responses, the present work started by assessing whether or not that different ability to induce cytokine release was a spurious fact or a characteristic of a given isolate. For this, a large set (n = 39) of PRRSV strains was isolated or retrieved from a collection and examined. To our knowledge no other study published before examined such a number of PRRSV isolates.

To categorise the isolates regarding their capabilities for inducing cytokine responses, four different cell systems were used: PAM, PBMC, BMDC and SwC3^**+ **^peripheral blood cells and three cytokines considered crucial in the development of the immune response were examined: IFN-α, IL-10 and TNF-α. Our study primarily revealed that every cell type has a different sensitivity in terms of the cytokine response against PRRSV and, as expected, the more specialised professional APC, BMDC and SwC3^**+ **^cells showed the greater ability to produce cytokines and SwC3^**+ **^cells were particularly highly sensitive. This difference between cell models indicates that responses of APC to PRRSV are also subjected to the idiosyncrasy of a particular cell type and emphasises the need to consider the system in which the results are obtained to have a correct interpretation of these cytokine readouts. Other authors [22] also reported that different types of DC have different susceptibilities to PRRSV and that they react differently to PRRSV infection. Therefore, the complexity of the in vivo response to the virus would be expected to reflect the heterogeneity of responses as well. In any case, examination of cell culture supernatants of BMDC or SwC3^**+ **^cells showed that IFN-α was not released upon PRRSV infection in accordance with previous reports using DC or PAM [7,22]. Interestingly, all four possible cytokine-inducing phenotypes for IL-10 and TNF-α were detected (from negative-negative to positive-positive) and, in most cases, differences between BMDC and SwC3^**+ **^cells were in the sense of a greater sensitivity of SwC3^**+ **^cells. Both cytokines, IL-10 and TNF-α, were produced as a result of the interaction with viable virus since inactivated virus did not induce them. Although the study of the mechanisms of regulation of cytokines was out of the scope of the present work, non structural PRRSV proteins could also be involved in this process. A recent paper [27] suggested that variations in TNF-α induction may be the result of variations in ORF-1a. These results might explain by themselves many of the discrepancies reported in the literature mentioned at the beginning of the present discussion.

The next step was to examine the effect of PRRSV infection in BMDC upon immunologically relevant cell surface molecules of BMDC. In general, the picture resulting from the flow cytometry observations did not support clear correlation between a given cytokine production pattern and the modification of different immunologically relevant cell surface markers. However strain 3267, which was unable to induce IFN-α, TNF-α or IL-10 release in any of the cell systems examined, produced extreme changes compared to the cytokine-inducing strains. Thus, strain 3267 induced the highest percentage of expression of SLA-II and CD80/86 and the lowest for CD163 and CD14. However, this increased expression of SLA-II and CD80/86 was not a co-expression in the same cells. Therefore, although increased, this could not lead necessarily to a more effective immune response. In fact, the lack of cytokine release upon a viral infection suggests a deep alteration of the expected response.

On the other side, the double positive strain (3262) induced the lowest percentage expression of CD80/86, the highest CD14 expression and a decreased percentage of cells expressed SLA-I compared to uninfected controls. To determine the role of IL-10, a blocking experiment was performed. That blocking of IL-10 restored SLA-I expression, partly restored CD80/86 expression, and reduced the expression of CD14; indicating that, at least, IL-10 was partially involved in the down-regulation of those molecules although, more than one regulation mechanism probably exists.

For CD163, the present results do not give a clear image of what happens with this molecule. Although some authors have described the ability of some strains to induce the expression of the viral co-receptor CD163 [28,29] which is known to be up-regulated by IL-10 [30], with our data, no correlation was found between the ability of the strain to induce IL-10 and the capability to enhance the expression of CD163.

In summary, the present paper shows that different PRRSV isolates may induce different patterns of cytokine release in APC, and may regulate differently the expression of immunologically relevant molecules. Also, it shows that IL-10 seems to play an important role in SLA-I, CD14 and probably CD80/86 but it is evident that this cytokine is not the only element in that regulation and, as some reports indicate [31,32] non-structural proteins seem to play an important role in the regulation of the innate responses against PRRSV. From a practical point of view, the present results also suggest that immunological studies of PRRSV cannot be performed with a single PRRSV strain if a global vision is desired or that genetic variability of PRRSV has to be taken into account when using clones or in vitro models.

## Competing interests

The authors declare that they have no competing interests.

## Authors' contributions

All authors participated in the design of the study, critical evaluation and discussion of the results and corrections to each version of the paper. Isolation, production and characterization of viruses were done by MG and EC. Obtention and culture of cells was carried out by MG, EC and LD. Phenotyping was done by MG, LD and ET. Cytokine ELISAs were developed and carried out by ID and MG. EM and MM directed and coordinated the present study. All authors read and approved the final manuscript.

## References

[B1] Loemba HD Mounir S Mardassi H Archambault D Dea S Kinetics of humoral immune response to the major structural proteins of the porcine reproductive and respiratory syndrome virus Arch Virol 1996 141 751 761 10.1007/BF01718333 8645111 PMC7086943

[B2] Díaz I Darwich L Pappaterra G Pujols J Mateu E Immune responses of pigs after experimental infection with a European strain of Porcine reproductive and respiratory syndrome virus J Gen Virol 2005 86 1943 1951 15958672 10.1099/vir.0.80959-0

[B3] Díaz I Darwich L Pappaterra G Pujols J Mateu E Different European-type vaccines against porcine reproductive and respiratory syndrome virus have different immunological properties and confer different protection to pigs Virology 2006 351 249 259 16712895 10.1016/j.virol.2006.03.046

[B4] Meier WA Galeota J Osorio FA Husmann RJ Schnitzlein WM Zuckermann FA Gradual development of the interferon-gamma response of swine to porcine reproductive and respiratory syndrome virus infection or vaccination Virology 2003 309 18 31 10.1016/S0042-6822(03)00009-6 12726723

[B5] Kimman TG Cornelissen LA Moormann RJ Rebel JM Stockhofe-Zurwieden N Challenges for porcine reproductive and respiratory syndrome virus (PRRSV) vaccinology Vaccine 2009 27 3704 3718 10.1016/j.vaccine.2009.04.022 19464553

[B6] Mateu E Diaz I The challenge of PRRS immunology Vet J 2008 177 345 351 10.1016/j.tvjl.2007.05.022 17644436 PMC7110845

[B7] Albina E Carrat C Charley B Interferon-alpha response to swine arterivirus (PoAV), the porcine reproductive and respiratory syndrome virus J Interferon Cytokine Res 1998 18 485 490 10.1089/jir.1998.18.485 9712364

[B8] Lee SM Schommer SK Kleiboeker SB Porcine reproductive and respiratory syndrome virus field isolates differ in in vitro interferon phenotypes Vet Immunol Immunopathol 2004 102 217 231 10.1016/j.vetimm.2004.09.009 15507307 PMC7112598

[B9] Charerntantanakul W Platt R Roth JA Effects of porcine reproductive and respiratory syndrome virus-infected antigen-presenting cells on T cell activation and antiviral cytokine production Viral Immunol 2006 19 646 661 10.1089/vim.2006.19.646 17201660

[B10] Park JY Kim HS Seo SH Characterization of interaction between porcine reproductive and respiratory syndrome virus and porcine dendritic cells J Microbiol Biotechnol 2008 18 1709 1716 18955824

[B11] Flores-Mendoza L Silva-Campa E Reséndiz M Osorio FA Hernández J Porcine reproductive and respiratory syndrome virus infects mature porcine dendritic cells and up-regulates interleukin-10 production Clin Vaccine Immunol 2008 15 720 725 10.1128/CVI.00224-07 18272667 PMC2292656

[B12] Peng YT Chaung HC Chang HL Chang HC Chung WB Modulations of phenotype and cytokine expression of porcine bone marrow-derived dendritic cells by porcine reproductive and respiratory syndrome virus Vet Microbiol 2009 136 359 365 10.1016/j.vetmic.2008.11.013 19128898

[B13] Silva-Campa E Cordoba L Fraile L Flores-Mendoza L Montoya M Hérnandez J Eureopean genotype of porcine reproductive and respiratory syndrome (PRRSV) infects monocyte-derived dendritic cells but does not induce Treg cells Virology 2010 396 264 271 10.1016/j.virol.2009.10.024 19913865

[B14] Martín M Segalés J Huang FF Guenette DK Mateu E de Deus N Meng XJ Association of hepatitis E virus (HEV) and postweaning multisystemic wasting syndrome (PMWS) with lesions of hepatitis in pigs Vet Microbiol 2007 122 16 24 17270366 10.1016/j.vetmic.2006.12.020

[B15] Quintana J Balasch M Segalés J Calsamiglia M Rodríguez-Arrioja GM Plana-Durán J Domingo M Experimental inoculation of porcine circoviruses type 1 (PCV1) and type 2 (PCV2) in rabbits and mice Vet Res 2002 33 229 237 10.1051/vetres:2002011 12056474

[B16] Segalés J Martínez-Guinó L Cortey M Navarro N Huerta E Sibila M Pujols J Kekarainen T Retrospective study on swine Torque teno virus genogroups 1 and 2 infection from 1985 to 2005 in Spain Vet Microbiol 2009 134 199 207 18814975 10.1016/j.vetmic.2008.08.002

[B17] Wensvoort G Terpstra C Pol JM ter Laak EA Bloemraad M de Kluyver EP Kragten C van Buiten L den Besten A Wagenaar F Broekhuijsen JM Moonen PLJM Zetstra T de Boer EA Tibben HJ de Jong MF van't Veld P Groenland GJR van Gennep JA Voets MTh Verheijden JHM Braamskamp J Mystery swine disease in the Netherlands: the isolation of Lelystad virus Vet Q 1991 13 121 130 1835211 10.1080/01652176.1991.9694296

[B18] Summerfield A McCullough KC Porcine bone marrow myeloid cells: phenotype and adhesion molecule expression J Leukoc Biol 1997 62 176 185 9261331 10.1002/jlb.62.2.176

[B19] Carrasco CP Rigden RC Schaffner R Gerber H Neuhaus V Inumaru S Takamatsu H Bertoni G McCullough KC Summerfield A Porcine dendritic cells generated in vitro: morphological, phenotypic and functional properties Immunology 2001 104 175 184 10.1046/j.1365-2567.2001.01299.x 11683958 PMC1783296

[B20] Guzylack-Piriou L Balmelli C McCullough KC Summerfield A Type-A CpG oligonucleotides activate exclusively porcine natural interferon-producing cells to secrete interferon-alpha, tumour necrosis factor-alpha and interleukin-12 Immunology 2004 112 28 37 10.1111/j.1365-2567.2004.01856.x 15096181 PMC1782461

[B21] Chang HC Peng YT Chang HL Chaung HC Chung WB Phenotypic and functional modulation of bone marrow-derived dendritic cells by porcine reproductive and respiratory syndrome virus Vet Microbiol 2008 129 281 293 10.1016/j.vetmic.2007.12.002 18221843

[B22] Loving CL Brockmeier SL Sacco RE Differential type I interferon activation and susceptibility of dendritic cell populations to porcine arterivirus Immunology 2007 120 217 229 10.1111/j.1365-2567.2006.02493.x 17116172 PMC2265861

[B23] Wang X Eaton M Mayer M Li H He D Nelson E Christopher-Hennings J Porcine reproductive and respiratory syndrome virus productively infects monocyte-derived dendritic cells and compromises their antigen-presenting ability Arch Virol 2007 152 289 303 10.1007/s00705-006-0857-1 17031757

[B24] Ait-Ali T Wilson AD Westcott DG Clapperton M Waterfall M Mellencamp MA Drew TW Bishop SC Archibald AL Innate immune responses to replication of porcine reproductive and respiratory syndrome virus in isolated Swine alveolar macrophages Viral Immunol 2007 20 105 18 10.1089/vim.2006.0078 17425425

[B25] Chiou MT Jeng CR Chueh LL Cheng CH Pang VF Effects of porcine reproductive and respiratory syndrome virus (isolate tw91) on porcine alveolar macrophages in vitro Vet Microbiol 2000 71 9 25 10.1016/S0378-1135(99)00159-5 10665530

[B26] López-Fuertes L Campos E Doménech N Ezquerra A Castro JM Domínguez J Alonso F Porcine reproductive and respiratory syndrome (PRRS) virus down-modulates TNF-alpha production in infected macrophages Virus Res 2000 69 41 46 10989184 10.1016/s0168-1702(00)00172-6

[B27] Gudmundsdottir I Risatti GR Infection of porcine alveolar macrophages with recombinant chimeric porcine reproductive and respiratory syndrome virus: effects on cellular gene transcription and virus growth Virus Res 2009 145 145 150 10.1016/j.virusres.2009.06.009 19540286

[B28] Calvert JG Slade DE Shields SL Jolie R Mannan RM Ankenbauer RG Welch SK CD163 expression confers susceptibility to porcine reproductive and respiratory syndrome viruses J Virol 2007 81 7371 7379 10.1128/JVI.00513-07 17494075 PMC1933360

[B29] Van Gorp H Van Breedam W Delputte PL Nauwynck HJ Sialoadhesin and CD163 join forces during entry of the porcine reproductive and respiratory syndrome virus J Gen Virol 2008 89 2943 2953 10.1099/vir.0.2008/005009-0 19008379

[B30] Patton JB Rowland RR Yoo D Chang KO Modulation of CD163 receptor expression and replication of porcine reproductive and respiratory syndrome virus in porcine macrophages Virus Res 2009 140 161 171 10.1016/j.virusres.2008.12.002 19111584

[B31] Chen Z Lauson S Sun Z Zhou X Guan X Cristopher-Hennings J Nelson EA Fang Y Identification of two auto-cleavage products of non-structural protein 1 (nsp1) in porcine reproductive and respiratory syndrome virus infected cells: nsp1 function as interferon antagonist Virology 2010 398 87 97 10.1016/j.virol.2009.11.033 20006994 PMC7111964

[B32] Kim DY Kaiser TJ Horlen K Keith ML Taylor LP Jolie R Calvert J Rowland RRR Insertion and deletion in a non-essential region of the nonstructural protein 2 (nsp2) of porcine reproductive and respiratory syndrome (PRRS) virus: effects on virulence and immunogenicity Virus Genes 2009 38 118 128 10.1007/s11262-008-0303-4 19048364

